# Physiological Degradation of Pectin in Papaya Cell Walls: Release of Long Chains Galacturonans Derived from Insoluble Fractions during Postharvest Fruit Ripening

**DOI:** 10.3389/fpls.2016.01120

**Published:** 2016-07-27

**Authors:** Samira B. R. do Prado, Paulo R. Melfi, Victor C. Castro-Alves, Sabrina G. Broetto, Elias S. Araújo, João R. O. do Nascimento, João P. Fabi

**Affiliations:** ^1^Department of Food Science and Experimental Nutrition, School of Pharmaceutical Sciences, University of São PauloSão Paulo, Brazil; ^2^University of São Paulo – NAPAN – Food and Nutrition Research CenterSão Paulo, Brazil; ^3^Food Research Center, CEPID-FAPESP (Research, Innovation and Dissemination Centers, São Paulo Research Foundation)São Paulo, Brazil

**Keywords:** papaya ripening, galacturonan, cell wall disassembly, pectin depolymerization, fruit softening

## Abstract

Papaya (*Carica papaya* L.) is a fleshy fruit that presents a rapid pulp softening during ripening. However, the timeline on how papaya pectinases act in polysaccharide solubilization and the consequent modification of the cell wall fractions during ripening is still not clear. In this work, the gene expression correlations between, on one hand, 16 enzymes potentially acting during papaya cell wall disassembling and, on the other hand, the monosaccharide composition of cell wall fractions during papaya ripening were evaluated. In order to explain differences in the ripening of papaya samplings, the molecular mass distribution of polysaccharides from water-soluble and oxalate-soluble fractions (WSF and OSF, respectively), as well as the oligosaccharide profiling from the WSF fraction, were evaluated by high performance size exclusion chromatography coupled to a refractive index detector and high performance anion-exchange chromatography coupled to pulse amperometric detection analyses, respectively. Results showed that up-regulated polygalacturonase and β-galactosidase genes were positively correlated with some monosaccharide profiles. In addition, an overall increase in the retention time of high molecular weight (HMW) and low molecular weight (LMW) polysaccharides in WSF and OSF was shown. The apparent disappearance of one HMW peak of the OSF may result from the conversion of pectin that were crosslinked with calcium into more soluble forms through the action of PGs, which would increase the solubilization of polysaccharides by lowering their molecular weight. Thus, the results allowed us to propose a detailed process of papaya cell wall disassembling that would affect sensorial properties and post-harvesting losses of this commercially important fruit.

## Introduction

The softening of fruit pulp is a major change that occurs during ripening, and pulp softening is mainly caused by cell wall disassembly (Gapper et al., 2013). The plant cell wall is composed of a matrix of cellulose microfibrils crosslinked to hemicelluloses and embedded with pectin, which also can be bound to cellulose (Carpita and Gibeaut, 1993; Ordaz-Ortiz et al., 2009; Gapper et al., 2013). Pulp softening is likely to occur by a reduction of cell-to-cell adhesion as a consequence of the dissolution of polysaccharides of the primary cell wall and middle lamella (Brummell and Harpster, 2001) by the action of hydrolases (Gapper et al., 2013; Balic et al., 2014). Different methods to identify those polysaccharides are based on plant cell wall fractionation, sugar composition, and molecular weight distribution to predict the possible biochemical modifications (Daher and Braybrook, 2015).

Papaya (*Carica papaya* L.) is a fleshy fruit that presents rapid pulp softening during ripening, which contributes to making the fruit edible but also increases post-harvest losses (Fabi et al., 2007). The softening of papaya fruit pulp is an ethylene-dependent process likely resulting from the action of several cell wall-related enzymes on the polysaccharide components of the plant cell wall and middle lamella. Previous works had indeed identified a critical subset of genes involved in cell-wall disassembly (Fabi et al., 2009, 2010, 2012). However, despite the relevance of this process to fruit quality, the role played by each enzyme, the polysaccharides affected, and the time course of the structural changes are not clear. Apparently, there is solubilization of large molecular mass galacturonans from pectins during ripening (Shiga et al., 2009); however, from which component of the cell wall the water-soluble galacturonans are derived and the degree of hydrolysis achieved remain elusive. At the same time, the up-regulation of PG, β-galactanases, and an endoxylanase have been associated with papaya softening (Fabi et al., 2014), but it is not clear how and when these enzymes act on the structural polysaccharides.

In this way, the present study aimed to investigate the correlations between 16 genes of cell wall-related enzymes identified in previous works (Fabi et al., 2009, 2010, 2012) and the changes in the monosaccharide composition of polysaccharides from the water-soluble, chelate-soluble, and ASFs of the cell wall during papaya ripening. As expected for other fleshy fruits, papaya WSF would correspond to the most soluble polysaccharides, including pectins, whereas the OSF would represent less soluble polysaccharides, mainly pectins that are tightened together by calcium bridges. On the other hand, the ASF would include celluloses, hemicelluloses, and even pectins bound to matrix glycans (Ordaz-Ortiz et al., 2009; Gapper et al., 2013). In addition, ripening-associated changes in molecular mass distribution of the water-soluble and chelate-soluble fractions and the presence of oligomers were investigated by size exclusion and anion-exchange chromatography. This is the first time a systematized mobilization of polysaccharides has been proposed in ‘Golden’ papaya pulp softening during ripening, and the cause of the cell wall disassembly caused by pectinase expression can open new perspectives on the mechanisms of papaya pulp softening.

## Materials and Methods

### Plant Material

Papaya fruits (*C. papaya* L. cv. ‘Golden’) were acquired from a producer in Aracruz (Espírito Santo, Brazil). Fruits were harvested from distinct plants at color break to one-fourth yellow (around 150 days post-anthesis) and were stored in 240-L chambers with controlled temperature and humidity (25 ± 0.1°C and 95%, respectively). Daily analyses were performed on, at least, six fruits until complete ripening. Carbon dioxide, ethylene, and pulp firmness were measured according to methods of Fabi et al. (2007). The fruits were individually placed in airtight-sealed jars and left at 25°C for 1 h. After that, air samples for ethylene and CO_2_ analysis (10 mL and 1 mL, respectively) were collected, and the composition of gasses was determined by gas chromatography using a flame ionization detector (FID) and a thermal conductivity detector (TCD) for ethylene and CO_2_ analysis, respectively (Agilent Technologies, model HP-6890). The column used was a HP-Plot Q (30 meters, I.D. 0.53 mm, Agilent Technologies) and the injector and detector temperatures were 250°C with an isothermal run at 30°C. Helium was used as gas carrier (1 mL min^-1^ for ethylene and 4 mL min^-1^ for CO_2_) and injections were performed in splitless pulse mode for ethylene and split mode for CO_2_ analysis (50:1). Ethylene and CO_2_ external standards in synthetic air (Air Liquid) were used for calibration curves. The remaining pulp following physiological analysis each day was pooled as two distinct biological replicates (at least three fruits each replicate), frozen in liquid N_2_, and stored at -80°C until analyses.

### Gene Expression Analyses of Papaya Cell Wall-Degrading Enzymes

Gene expression of papaya cell wall-degrading enzymes were measured according to methods of Fabi et al. (2014) and following the “Minimum Information for Publication of Quantitative Real-Time PCR Experiments – MIQE” (Bustin et al., 2009). The primer sequences for genes are depicted in **Supplementary Table S1** (PG – *PG1* and *PG2*; *PG* 3 “QRT2” – *PG3*; PL – *PL1*; PL family – *PL2*; AGAL – *AGAL1* and *AGAL3*; BGAL – *BGAL1* and *BGAL3*; PME – *PME1*, *PME2*, and *PME3*; ARF – *ARF*; XYL – *XYL*; CELL – *CELL*; and XTH [ext/EXGT-A1] – *XTH*). Internal controls (reference genes) were the actin (*ACT*), the elongation factor 1-α (*EF1*), and the ubiquitin (*UBQ*) genes, as previously reported (Fabi et al., 2014; Broetto et al., 2015), and RNA expression levels did not influence data, as suggested by the GeNorm analyses (Vandesompele et al., 2002). The geometrical means of the threshold cycle (Ct) values were achieved for the analysis of relative expression (Vandesompele et al., 2002). Expressions of one new gene for an α-galactosidase (*AGAL3*, located on chromosome LG7 contig 16621 – GenBank accession number **ABIM01016598.1**) and another gene for a β-galactosidase (*BGAL3*, located on chromosome Un contig 26518 – GenBank accession number **ABIM01026480.1**) were also analyzed. All amplicons were sub-cloned and sequenced in order to confirm gene identity. Real-time PCR was performed using a four-channel Rotor-Gene 3000 multiplexing system (Corbett Research, Sydney, NSW, Australia). The melting curves of amplicons and non-template controls were continuously checked in all experiments. The Ct values (four technical replicates from two biological replicates) were computed using the Rotor-Gene 3000 software, and quantification was performed using the relative standard curve method (Pfaffl, 2001). Results of the standard curves calculations are shown in **Supplementary Table S3**.

### Papaya Cell Wall Polysaccharide Extraction

Polysaccharide extraction scheme is summarized in **Supplementary Figure S1**.

#### Total Cell Wall Polysaccharides

The frozen papaya pulp was triturated and extracted three times with chloroform:methanol (1:1, v/v) for enzyme inactivation and protein/pigment removal. Residues were washed with three volumes of 80% boiling ethanol for monosaccharide removal and were also washed with three volumes of acetone for drying purposes. Finally, residues were dried and weighed, resulting in a TCW.

#### Water-Soluble, Oxalate-Soluble, and Alkali-Soluble Cell Wall Polysaccharides

The TCW were extracted three times with 20 mL deionized water under constant magnetic stirring for 20 min at 25°C and centrifuged (10000 × *g*, 20 min, 25°C). The supernatant, or WSF, was lyophilized and weighed. Residues of WSF were extracted according to methods of Taboada et al. (2010), with modifications. Briefly, residues were extracted three times with 150 mL of 0.08 M (NH_4_)_2_C_2_O_4_⋅H_2_O (pH 4.5) under constant magnetic stirring for 30 min at 25°C and centrifuged (1500 × *g*, 10 min, 25°C). The supernatant, or OSF, was dialyzed against continuously replaced distilled water for 3 days using Millipore dialysis membranes (MWCO 3.5 kDa; Billerica, MA, USA), lyophilized, and weighed.

Finally, remnant residues of WSF and OSF were extracted three times with 4 M NaOH and 0.2 M NaBH_4_ under an N_2_ stream and constant magnetic stirring overnight at 25°C and centrifuged (1500 × *g*, 10 min, 25°C). The supernatant, or ASF, was dialyzed against continuously replaced distilled water for 3 days using Millipore dialysis membranes (MWCO 3.5 kDa), lyophilized, and weighed. The remnant, or IF, was lyophilized and weighed. The yield was calculated for all fractions in relation (%) to papaya pulp fresh weight.

### Papaya Cell Wall Polysaccharide Composition

Hydrolyzed monosaccharides were generated by trifluoroacetic acid and H_2_SO_4_ hydrolysis (Shiga et al., 2009). The supernatants obtained were analyzed for neutral sugars and uronic acids by HPAEC-PAD according to methods of Shiga et al. (2009). Briefly, 1 mg of polysaccharides obtained by extractions (WSF and OSF) was hydrolyzed with 1 mL of 2 M TFA at 120°C for 60 min in a screw-capped conical vial and centrifuged (2000 × *g*, 5 min, 25°C). Supernatants were transferred to new vials, dried under an N_2_ stream, and separated for analysis. The same procedure was applied for ASF and IF, but the precipitates that resulted from TFA hydrolysis (the cellulose-rich residues) were dried under an N_2_ stream and rehydrolyzed with 0.9 mL of 2 M H_2_SO_4_ at 120°C for 90 min. After hydrolysis, supernatants were neutralized with 0.1 mL of 50% NaOH (w/w) and analyzed in a DX 500 HPAEC-PAD system (Dionex, Sunnyvale, CA, USA). Neutral sugars (L-arabinose, D-galactose, D-glucose, D-fucose, D-mannose, L-rhamnose, and D-xylose) and uronic acids (D-glucuronic and D-galacturonic acid) were used as standards (Sigma; St. Louis, MO, USA).

### Papaya Cell Wall Polysaccharide Homogeneity and Molecular Weight

Molecular mass distribution of the papaya polysaccharides was analyzed by HPSEC-RID via a 1250 Infinity system (Agilent, Santa Clara, CA, USA). Samples were diluted with water (0.5 mg/mL), injected (25 μL) and separation was conducted through two PL aquagel-OH MIXED-M (300 mm × 7.5 mm, 8 μm) columns (Agilent). The eluent was 0.2 M NaNO_3_ at 35°C with a flow of 0.6 mL/min. Molecular weights were estimated using dextran T-series (5, 12, 25, 50, 80, 150, 410, and 750 kDa) (Sigma; St. Louis, MO, USA) as external standards.

### Papaya Cell Wall Oligosaccharide Profiling

The low molecular weight peaks from WSF at 1 and 5 DAH were separated by ultrafiltration using Millipore Amicon Ultra-4 centrifugal filter units (MWCO 30 kDa). The oligosaccharide profiles were analyzed using a DX 500 HPAEC-PAD system (Dionex, Sunnyvalle, CA, USA) as described by Jonathan et al. (2012). Briefly, samples were diluted in 500 μL of water, injected (25 μL) and their profiles were analyzed in a CarboPac PA-1 column (2 mm × 250 mm) (Dionex). Oligomers derived from neutral sugars were eluted (0.3 mL/min) with a linear gradient of 0.02–0.05 M NaOH for 3 min and 0.05–0.075 M NaOH for 10 min, followed by isocratic elution of 0.1 M NaOH for 2 min. Oligomers derived from uronic acids were then eluted with a gradient of 0–1 M NaOAc in 0.1 M NaOH for 50 min. Finally, the column was washed with 1 M NaOAc in 0.1 M NaOH for 7 min followed by 0.1 M NaOH for 3 min. Equilibration was done by eluting 0.02 M NaOH for 20 min. Standards sugars (L-arabinose, D-galactose, D-glucose, D-fucose, D-mannose, L-rhamnose, D-xylose, D-galacturonic acid, and digalacturonic acid) and oligosaccharides (maltotriose, maltopentaose, maltohexaose, and trigalacturonic acid) were used as external standards (Sigma).

### Statistics

The results were expressed as the mean ± standard deviation (SD) obtained from, at least, two technical and two biological replicates. We did not conduct an analysis of variance since it would not lead to statistically valid results (low number of biological replicates). Data were analyzed using GraphPad Prism version 6.0 software (GraphPad Software, San Diego, CA, USA). To perform Pearson linear correlations and PCA data were transformed by log, to obtain homogeneity of variance. The Pearson correlation coefficients were calculated using the quadruplicates of genes and monosaccharide values and the heat maps were done in GENE-E version 3.0.204 (Broad Institute, Inc., Cambridge, MA, USA). Twenty five variables were submitted to a PCA, adopting the genes and monosaccharide as columns and the 5 DAH, in quadruplicate, as rows. Eigenvalues higher than 1.0 were adopted to explain the projection of the samples on the two-dimensional graph.

## Results

**FIGURE 1:**
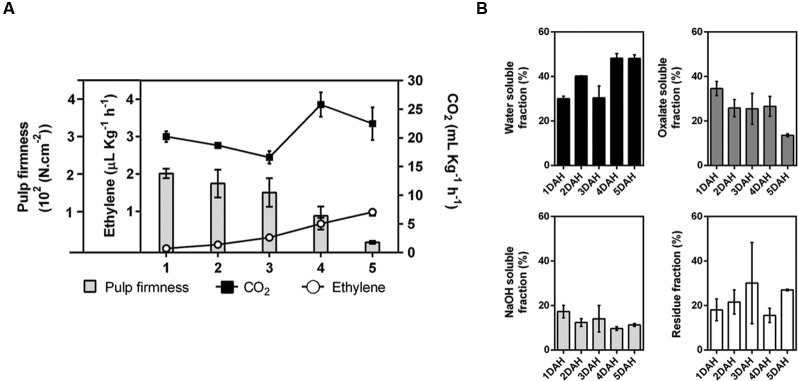
**Ripening of papaya fruit.** The amount of CO_2_ produced by respiration (black squares – mg.Kg^-1^.h^-1^), the production of ethylene (open circles – μL.Kg^-1^.h^-1^), and the pulp firmness (bars – 10^2^(N.cm^-2^)) were examined during ripening. Error bars indicate SDs of the mean for, at least, six fruits (*n* = 6) **(A)**. Yield of cell wall fractions in the pulp of ripening papayas, based on fresh weight basis (percentage of each cell wall fraction using total cell wall as 100%) **(B)**. Standard deviation values (duplicate for each biological replicate, *n* = 4) are depicted in each corresponding cell wall fraction bars. DAH: days after harvesting.

During the 5 DAH, there was appreciable softening of the pulp of papayas (**Figure 1A**). Although the extraction yield of total cell wall material from the fruit pulp appeared not changed during ripening, the specific yields of some fractions were quite different, as the WSF and IF clearly increased through ripening, whereas the OSF and ASF decreased (**Figure 1B**).

**FIGURE 2:**
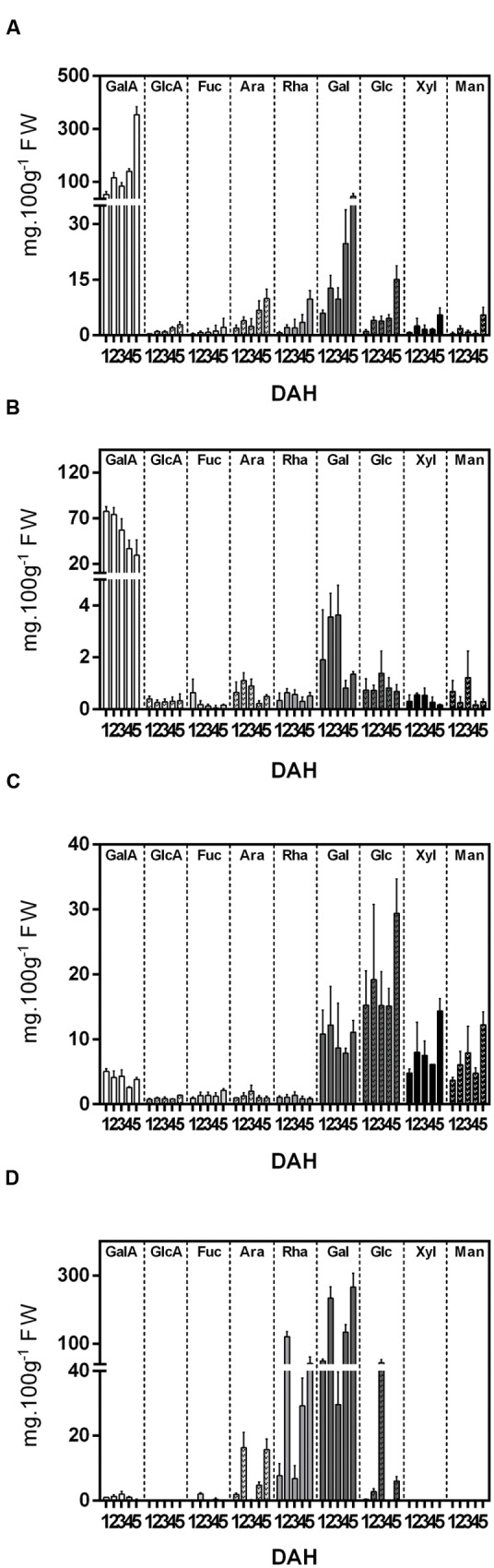
**Monosaccharide composition (mg.100g-1 Fresh Weight) of cell wall fractions isolated from papaya during 5 DAH.** **(A)** Water Soluble Fraction (WSF); **(B)** Oxalate Soluble Fraction (OSF); **(C)** Alkali Soluble Fraction; **(D)** Insoluble fraction. Galacturonic acid (GalA), glucuronic acid (GlcA), fucose (Fuc), arabinose (Ara), rhamnose (Rha), galactose (Gal), glucose (Glc), xylose (Xyl), mannose (Man). Error bars indicate SDs of the mean (duplicate for each biological replicate, *n* = 4).

The monosaccharide composition of the cell wall fractions was investigated and revealed GalA, followed by Gal, was the most abundant monosaccharide in the WSF and OSF. In contrast, neutral sugars, such as Gal, Glc, Man, and Xyl, predominated in the ASF, while Gal and Rha were more abundant in the IF (**Figure 2**). There was an overall increase in acid and neutral sugars in the WSF during ripening, which was inversely correlated to a monosaccharide decrease in the OSF.

**FIGURE 3:**
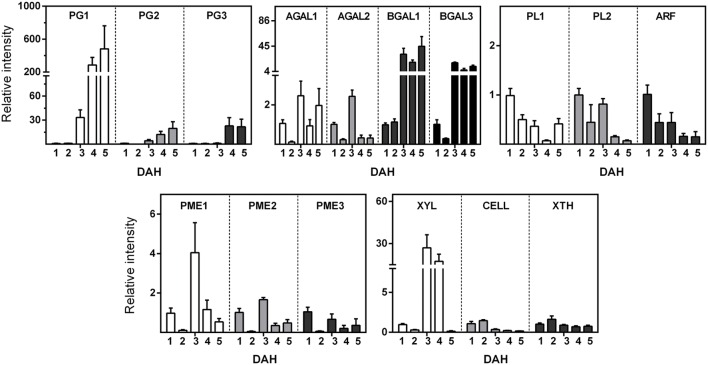
**Gene Expression of enzymes related to cell wall disassembling during papaya ripening.** Real-time PCR (qPCR) was used to analyze the mRNA levels of various genes during ripening. The column heights indicate the relative mRNA abundance; the expression values for unripen fruit (first day after harvesting) were set to 1. The error bars on each column indicate the SD of four technical replicates for each biological replicate (*n* = 8). Polygalacturonase 1 (PG1); Polygalacturonase 2 (PG2); Polygalacturonase 3 “QRT2” (PG3); Pectate lyase (PL1); Pectate lyase family (PL2); Alpha-galactosidase (AGAL1); Alpha-galactosidase (AGAL3); Beta-galactosidase (BGAL1); Beta-galactosidase (BGAL3); Pectinesterase (pectin methylesterase; PME1); Pectinesterase (pectin methylesterase; PME2); Pectinesterase (pectin methylesterase; PME3); Alpha-L-arabinofuranosidase (ARF); Xylan endohydrolase (XYL); Cellulase (CELL); and Xyloglucan endotransglycosylase (ext/EXGT-A1; XTH). The primer sequences and GenBank accession numbers for genes are depicted in **Supplementary Tables S1** and **S2**.

The expression analysis of 16 cell wall-related enzymes (**Figure 3**) showed different up-regulated and down-regulated genes during papaya ripening. Interestingly, *PG*, galactosidase, and *XYL* genes showed the most significant changes in relative expression. The *PGs* and *BGAL1* seemed to increase during ripening, while the *ARF* and *PLs* seemed to decrease. It was noteworthy that *BGAL1* and *BGAL3* increased after day 2 and maintained higher values comparing with day 1 and 2. Generally, *PL1*, *PL2*, and *ARF* showed lower values starting from day 2. *PME1* and *PME2* expression peaked at day 3, and *XYL* peaked at day 3 with higher values even at day 4.

**FIGURE 4:**
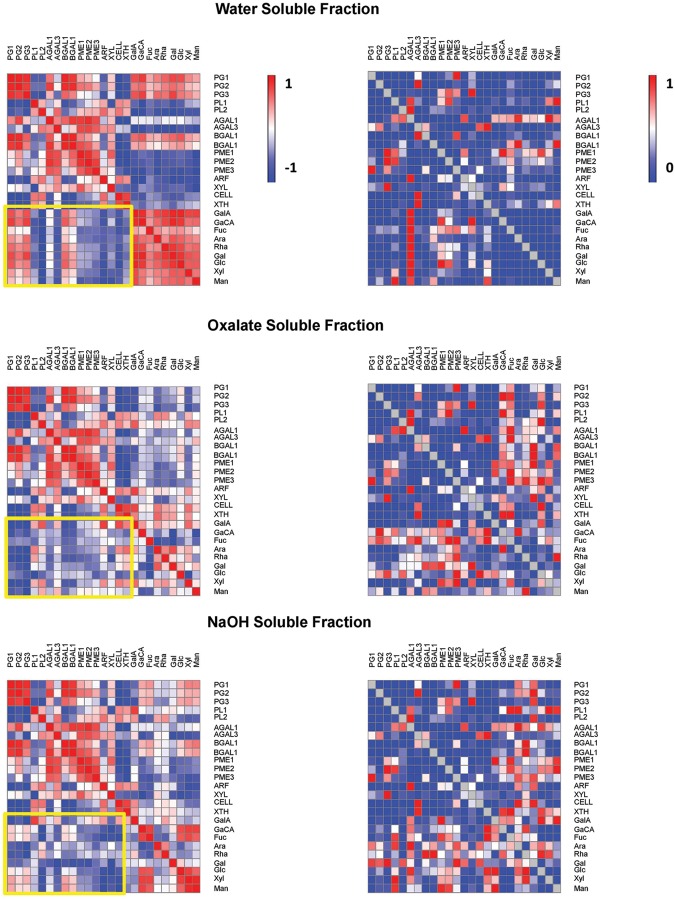
**Pearson correlations and associated *p*-values of papaya cell wall-related genes and monosaccharides.** Pearson correlation of papaya cell wall-related genes and monosaccharides cell wall composition from WSF, OSF, and ASF were analyzed. SPSS software was used to calculate the Pearson correlation and the corresponding *p*-values for the 25 combinations using the 16 values of gene expression and 9 values of monosaccharides. **(Left)** in figure are the heat maps of correlation values described as positive values that were set to red color and negative values that were set to blue color. **(Right)** in figure are the heat maps of *p* values as values near to one that were set to red color and values near to zero that were set to blue color.

A correlation matrix was obtained for the gene expression and the monosaccharides of WSF, OSF, and ASF during ripening (**Figure 4**). Overall, *PGs* and *BGALs* were positively correlated to uronic acids and most of the neutral sugars in the WSF but were negatively correlated in the OSF. Negative correlations of *PGs* and *BGALs* were also noticed in the ASF but were limited to GalA, Gal, Ara, and Rha. Regarding *XYL*, which was also significantly up-regulated, negative correlations with uronic acids and neutral sugars were observed in the WSF and ASF, especially Gal, Glc, and GlcA.

**FIGURE 5:**
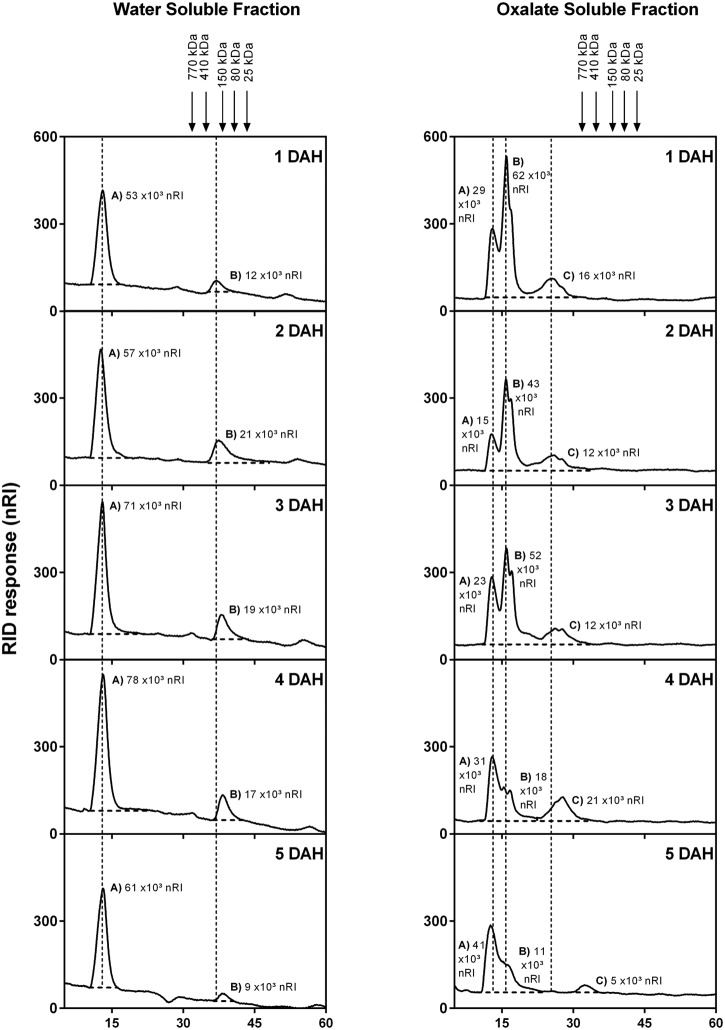
**High performance size exclusion chromatography-RID elution profile of papaya WSFs for 1 to 5 DAH.** HPSEC coupled to a refractive index detector was used to evaluate the molecular weight distribution in WSF and OSF extracted from total cell wall obtained from papaya pulp. Molecular weights were estimated using a standard curve of dextran T-series (25, 80, 150, 410, and 750 kDa) showed with arrows in the figure. The peaks were marked with dotted lines in order to facilitate retention time comparisons. Peaks were divided in (A,B) (for WSF) and (A–C) (For OSF), and total area values are depicted in graphics. DAH: days after harvesting.

**FIGURE 6:**
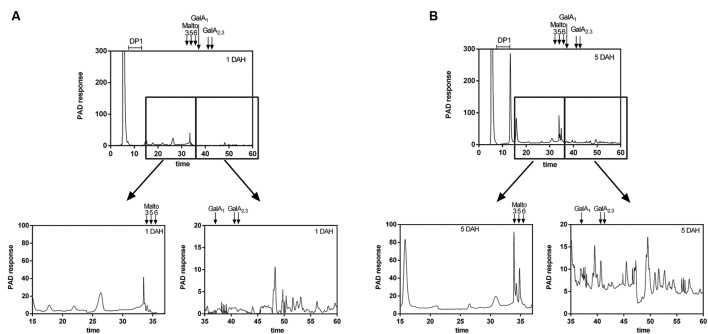
**High Performance Anion Exchange Chromatography (HPAEC) elution profile of papaya oligosaccharides smaller than 30 kDa at 1 and 5 DAH (days after harvest).** The low molecular weight oligosaccharides from WSF at 1 DAH **(A)** and 5 DAH **(B)** were separated by ultrafiltration (<30 kDa) using Millipore Amicon Ultra-4 centrifugal filter units and analyzed using a DX 500 HPAEC coupled with Pulsed Electrochemical Detection (Dionex) in a CarboPac PA-1 column. Oligos derived from neutral sugars were eluted first using a NaOH gradient, while oligos derived from uronic acids were later eluted using NaOAc/NaOH gradient. Maltotriose, maltopentaose, and maltohexaose, as well as mono, di and tri-galacturonic acid DAH, days after harvesting.

The homogeneity and molecular weight distribution of WSF and OSF during papaya ripening were monitored by HPSEC-RID (**Figure 5**). In the chromatograms of the WSF, the peak area corresponding to HMWP increased during ripening, while a second peak of LMWP diminished, the same time during which retention time slightly increased. The chromatographic separation of the OSF revealed three peaks, and two of them were significantly affected during ripening. The main peak corresponding to HMWP almost disappeared, while the retention time of the peak of LMWP remarkably increased. The WSF from fruit sampled at day 1 and day 5 were also examined for the presence of oligosaccharides using HPAEC-PAD (**Figure 6**), and the chromatograms revealed an increase in the diversity and abundance of oligosaccharides derived from both neutral and acidic sugars at 5 DAH.

**FIGURE 7:**
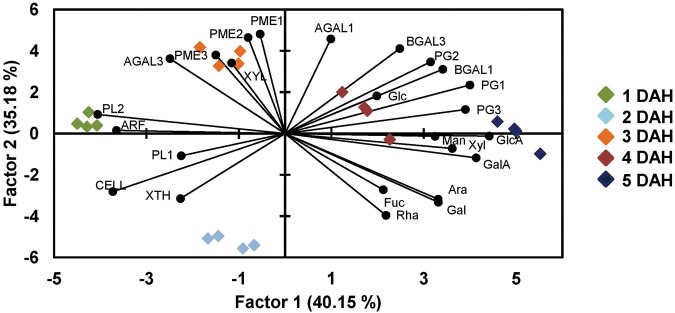
**Principal component analysis of papaya samples analysis comparing monosaccharides and genes expression.** A PCA analysis was done in order to distinguish the uppermost differences in papaya samples for days from 1 to 5 after harvesting using data from sugars composition and genes expression. PCA revealed that factors 1 and 2 accounted for more than 75% of the variability and separating papaya fruits sampled at 1, 2, and 3 DAH from those at 4 and 5 DAH. The high correlations of PGs and BGALs to papaya at 5 DAH and of PMEs, XYL, and AGAL3 to fruits at 3 DAH were the highest observed discrepancies.

PCA of the whole dataset revealed that factors 1 and 2 accounted for more than 75% of the variability (**Figure 7**). The combination of Factor 1 and 2 allowed the discrimination between all 5 sampling days. Factor 1 allowed the separation of papaya fruit samples at 1 DAH (quadrant on the left) from 5 DAH (quadrants on the right). GlcA, GalA, PG1, and *PG3* were the strongest variables that forced day 5 to get on the right side in the graphic. *PL2* and *ARF* forced day 1 to get on the left size. Day 3 correlates with *AGAL2*, *PME1*, and *PME2* and – less strongly – with *XYL* and *PME3*. Day 4 correlates with Glc and Day 5 with GlcA.

## Discussion

In general, climacteric fruit ripening is fast due to ethylene biosynthesis and its self-regulation (Gapper et al., 2013). The triggering of ethylene-dependent biochemical reactions in papaya ripening, such as pulp softening, affects fruit quality and post-harvest handling (Fabi et al., 2007). Previous works had identified a subset of genes involved in cell-wall disassembly (Fabi et al., 2009, 2010, 2012) that were studied in the present work. Despite the importance, it was not clear how and when cell wall-related enzymes act in the solubilization and modification of papaya cell wall fractions. In this way, the following discussion attempted to fill in those gaps.

### Polygalacturonases and Galactanases Are the Main Factors Responsible for Galacturonan Depolymerization in Papaya Pulp

Differences in cell wall yield, monosaccharide composition from fractions, and gene expression of cell wall-related enzymes in papayas can provide evidence regarding pectin molecule structure in papaya pulp. These structures would be more or less disposable for biochemical changes that lead to a determined phenotypic characteristic in a short period of time (soft pulp in ripe fruit). According to the analysis of the papaya cell wall fractions, the softening of the fruit pulp was followed by an apparent increase in the yield of water-soluble polysaccharides. The increase in acid and neutral sugars in the WSF, especially GalA, was inversely correlated to the respective monosaccharide decrease in the OSF, suggesting a massive release of less soluble galacturonans during papaya ripening. Since the changes in Ara, Rha, and Gal contents were similar to that of GalA, it seems that there was solubilization of rhamnogalacturonan I portions of the OSF. The Gal increase in the WSF also would suggest the solubilization of galactans from the OSF during ripening.

The up-regulation of *PG1*, *PG2*, *PG3*, and *XYL* genes, which have been associated previously with papaya pectin disassembly (Fabi et al., 2014), could explain some important changes in monosaccharide composition of the cell wall fractions. The up-regulation of *PGs* and *XYL* has been correlated to the release of GalA and Xyl to the WSF, reinforcing the idea of a massive hydrolysis of galacturonans and xylans from the OSF and ASF, respectively, during papaya ripening. The combined action of up-regulated *PGs* and *XYL*, as well as *XTH* (slightly increase on day 2), would agree with the proposal of Lazan et al. (2004), who suggested the presence of xyloglucan–pectin linkages in papaya. The disruption of those linkages during ripening would lead to solubilization of the pectin tightly bound to more IF of the papaya cell wall.

The action of *PGs* on homogalacturonans and the action of *GALs* on the side chains could have contributed to solubilization of rhamnogalacturonan I portions of the pectins, but not the action of *PLs* nor *ARF*. The expression of galactosidases has been associated with papaya pulp softening (Lazan et al., 2004; Soh et al., 2006; Fabi et al., 2014), and the present research revealed up-regulation of β*-GALs*, including a newly identified β*-GAL* gene (β*-GAL3*). *A-GALs* had also an apparent up-regulation but not at the higher levels as β*-GALs*, which might have assisted for the significant release of Gal during fruit ripening. Similar biochemical changes were described for ‘Jonagold’ apple, since increases in galactosidase enzyme activity promoted losses of Gal from the side chains of rhamnogalacturonan I, leading to earlier softening during low temperature storage (Gwanpua et al., 2014). The presence of GalA in the ASF denoted the occurrence of pectin firmly bound to the IF of the cell wall via the side chains and/or backbone (Zykwinska et al., 2005; Brummell, 2006). Regarding the fact that methylation of WSF increases during papaya ripening (Manrique and Lajolo, 2004; Shiga et al., 2009), the up-regulation of *PMEs* could be related to an increasing in calcium bridges by demethylation that allowed pectins to be continuously tightly bound to the IF besides *PGs* depolimerization (Wang et al., 2015).

The increase in Glc amounts in all fractions during ripening (WSF, OSF, and ASF) was noteworthy, which could result from the solubilization of hemicelluloses tightly bound to small pieces of cellulose by the action of *XTH* and *XYL*, making more soluble complexes. Although expression of the *CELL* gene is not suggestive of a relevant role in papaya cell wall degradation, the action of other up-regulated CELLs that have not been identified may not be disregarded. The PCA analysis provided an integrated view of the changes in gene expression and the monosaccharide composition of the cell wall fractions, evidencing the positive correlations between *PGs* and *BGALs* and between *PMEs* and *XYL* to the changes in neutral and acid sugars during papaya ripening.

### Mobilization of High Molecular Weight Polysaccharides from Insoluble to Soluble Fractions is Responsible for Papaya Pulp Softening

The decreasing pattern in the OSF and the increasing one in the WSF for GalA, Fuc, Ara, Rha, and Gal quantities suggest that a massive migration of insoluble pectins to more soluble forms occurred during papaya ripening. The correlation values between sugar quantities and cell wall-related gene expression also could support this suggestion, since the pectin molecular weight would decrease due to pectinase action.

The study of polysaccharides by HPSEC-RID analysis showed an overall increase in the retention time of HMW and LMW polysaccharides, which could be attributed to the depolymerizing action of pectinases, mainly PG, in both the WSF and OSF. The apparent disappearance of one HMW peak of the OSF may result from the conversion of pectins that were crosslinked with calcium into more soluble forms through the action of *PGs*, which would increase the solubilization of polysaccharides by lowering their molecular weight. Therefore, the pectin solubilizing flow from the insoluble parts of papaya cell walls would contribute to papaya softening. In this process, the release of insoluble pectins tightly bound to celluloses, xyloglucans, and xylans would enrich the OSF (Zykwinska et al., 2008; Cosgrove, 2014; Wang et al., 2015), and the continuous depolymerization of pectins tightly bound to each other by calcium bridges (insoluble) would enrich the WSF, making cell wall adhesion weaker and causing tissue softening. Based on the gene expression analysis, it is likely the highly expressed pectinases would act on pectins tightly bound to xyloglucans and xylans (Popper and Fry, 2008) that had been depolymerized partially by *XYL* and *XTH* (Manenoi and Paull, 2007; Han et al., 2015), leading to disruption of cell walls. A similar process had been reported for long-storage carrots, in which the WSF was enriched by degraded polysaccharides from the chelate-soluble fraction due to enzymatic degradation and solubilization of polysaccharides from chelate-soluble and ASFs (Cybulska et al., 2015).

In addition to the release of soluble polysaccharide chains from the cell wall, the massive depolymerization of pectins resulted in high quantities of pectin-derived oligomers in the WSF at day 5, as previously observed for ‘Maradol’ papayas (Sañudo-Barajas et al., 2009). The HPAEC-PAD analysis of LMW peaks eluted from HPSEC-RID showed the oligomers had a mixed profile of neutral and acidic sugars. Some oligomers were composed of neutral sugars, and since the technique cannot distinguish between linear and branched oligomers, the detected oligomers may have derived from rhamnogalacturonans. The predominance of peaks eluted after the trigalacturonic acid standard may indicate the release of some oligomers from homogalacturonans by the action of endopolygalacturonases.

In general, climacteric fruits present the expression of PGs as the key role in fruit softening despite it is not the only enzyme responsible for this process. Papayas seem to express high quantities of PGs transcripts during ripening when compared to other plant species (Brummell, 2006) and this is due to variation in the composition of cell walls and the different rate of pulp softening (Prasanna et al., 2007). Papaya contains high molecular weight galacturonans, that probably require high quantities of *PGs* for a rapid pulp softening by solubilizing homogalacturonans and concomitant up-regulation of β*-GALs* could have contributed to solubilization of rhamnogalacturonan I. Apples had higher PGs activities only late in ripening and the high β-Gal was a very important event during early ripening (Gwanpua et al., 2014). For carrots the mechanisms responsible for the softening may be the disintegration of the network of more IF into shortest molecules (action of β-Gal, α-L-Af and PGs enzymes) and the polymer size increased in WSP (Cybulska et al., 2015).

As expected for future works, gene silencing of the three *PGs* genes from papaya (*PG1*, *PG2*, and *PG3*) could abolish the release of homogalacturonan long chains during postharvest fruit ripening showing a central role of PGs on pulp softening, unless other set of genes would be highly expressed and responsible for marginal pulp softening (which seems not to be the case of). The study of the cell-wall degrading enzyme activities such as PGs, GALs, and XYLs, that had mRNA levels highly altered during fruit ripening, would reinforce the releasing of galacturonans during papaya ripening. However, one could argue enzyme activity experiments would not account for the expression of specific genes, so it would be important to correlate with gene expression data (Fabi et al., 2014).

The softening of papaya fruit during ripening is a relatively fast and complex process likely resulting from the coordinated action of several enzymes on the polysaccharide structure of the plant cell wall. In this regard, the results of the present research point to the action of pectinases, mainly PG and galactanases, as well as a xylanase as being responsible for the mobilization of HMW pectins from less soluble to more soluble fractions, especially the pectins tightly bound to cellulose/hemicellulose and to each other by calcium bridges. In this way, understanding the biochemical pathways that lead to papaya pulp softening would be of valuable interest, since the fruit could be represented as a model for *in vivo* rapid cell wall polysaccharide solubilization that instantly alters sensorial properties and post-harvesting losses of this commercially important fruit.

## Author Contributions

Conceived and designed the experiments: JF. Performed the experiments: SdP, PM, SB, VC-A, EA. Analyzed the data: SdP. Wrote the paper: SdP, JF. Supervised work: JF. Revised critically and finalized the manuscript: JdN, JF.

## Conflict of Interest Statement

The authors declare that the research was conducted in the absence of any commercial or financial relationships that could be construed as a potential conflict of interest.

The reviewer AG and handling Editor declared their shared affiliation, and the handling Editor states that the process nevertheless met the standards of a fair and objective review.

## Abbreviations

AGAL: α-galactosidases
ASF: alkali-soluble fraction
ARF: α-L-arabinofuranosidase
Ara: arabinose
BGAL: β-galactosidases
CELL: cellulase
DAH: days after harvesting
Fuc: fucose
GalA: galacturonic acid
GlcA: glucuronic acid
Gal: galactose
Glc: glucose
HPSEC-RID: high performance size exclusion chromatography coupled to a refractive index detector
HPAEC-PAD: high performance anion-exchange chromatography coupled to pulse amperometric detector
HMWP: high molecular weight polysaccharides
IF: insoluble fraction
LMWP: low molecular weight polysaccharides
Man: mannose
OSF: oxalate-soluble fraction
PCA: Principal component analysis
PG: polygalacturonases
PL: pectate lyase
PME: pectinesterases
Rha: rhamnose
TCW: total cell wall fraction
WSF: water-soluble fraction
Xyl: xylose
XYL: xylan endohydrolase
XTH: xyloglucan endotransglycosylase
