# Supplementary material for: Physiological Degradation of Pectin in Papaya Cell Walls: Release of Long Chains Galacturonans Derived from Insoluble Fractions during Postharvest Fruit Ripening
Source: Front Plant Sci. 2016 Jul 27;7:1120. doi: 10.3389/fpls.2016.01120 (PMC4961711; doi:10.3389/fpls.2016.01120)
Supplement: Supplementary file 1 [file Table_1.PDF]

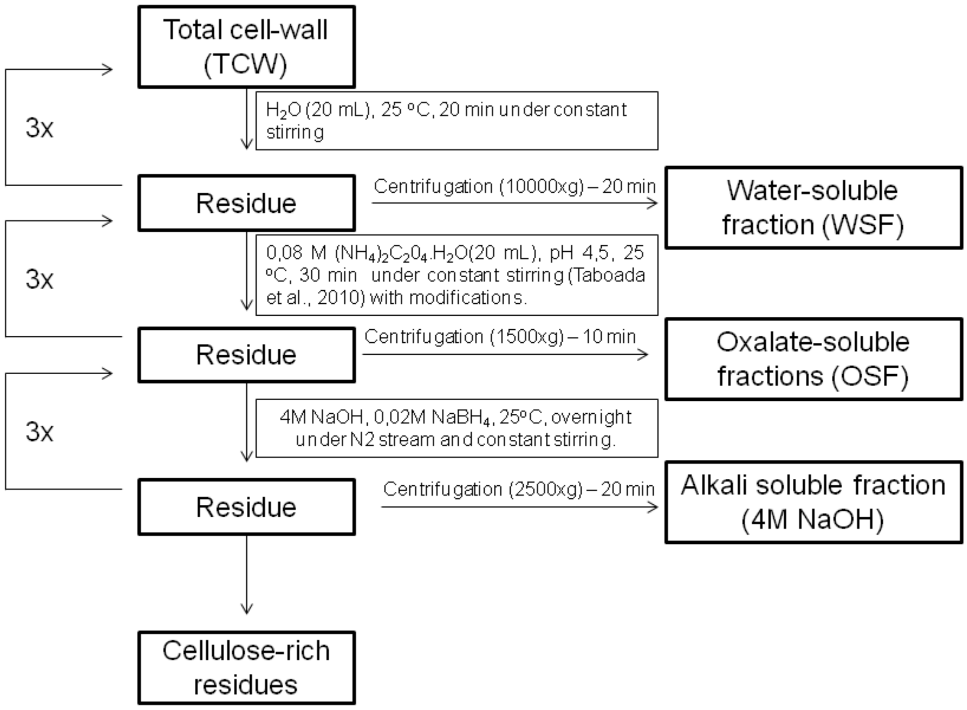

Fig. S1. Schematic sequence of papaya cell wall extraction and fractionation.

Table S1. Nucleotide sequences used in qPCR.

Gene	*Primer name*	*Sequence (5'→3')*
cpPG1	>pg1_f	TGG TGG TGC GTA TAG ATG GA
	>pg1_r	ACA AAA CCC AGT ACC CAC CA
cpPG2	>pg2_f	TCC TGA AGC TCA CCC TTC AT
	>pg2_r	CCT CAA TGC CTT TGA AGC TC
cpPG3	>pg3_f	TTG GAG GGC AGC TTG TTT AG
	>pg3_r	CAC CCA AGC CTT TAT TGT TCC
cpPL1	>pl1_f	TTC CCT GTG GGC TTA CAA TC
	>pl1_r	AGG GAG GTC TGC CAT TAC AT
cpPL2	>pl2_f	CAT GTT CTT GTC CTG CGT GT
	>pl2_r	CCA TCC ACG GCT CTA ATT TC
cpAGAL1	>a-gal1_f	GTA TAG GCG GAA GGT GGA T
	>a-gal1_r	AAG GCC ACC TCT CTG GAT
cpAGAL3	>a-gal3_f	TGT CCG TTG ATC CTT CAG TG
	>a-gal3_r	AGT CCG GAA GAA TGC TGA TG
cpBGAL1	>bgal1_f	GTG CTT GCA ACT ATG CTG GA
	>bgal1_r	ATA GGT TCG CAG TTG GGT TG
cpBGAL3	>bgal3_f	CCA AAG TGG GGA CAT TTG AG
	>bgal3_r	ACA CCC AGA CTT CGA CTT G
cpPME1	>pme1_f	TAT CTT GGT AGG CCC TGG A
	>pme1_r	AGG CCA GTG TTT CGG TAC T
cpPME2	>pme2_f	GTG GTT TGT TCC TCA GCA CA
	>pme2_r	TGG AAC GTA ACT GCA AGT GG
cpPME3	>pme3_f	GCA AGC TTT AGG GGT GTT GA
	>pme3_r	AGG CCT GCA GAG CTT ATT GA
cpARF	>arf_f	AGG TGG CTG TTT TGT TGA GG
	>arf_r	TCT CTT CCC AAG GTC CAA TG
cpXYL	>xyl_f	GCT TCC GCT GTG TTT TAT GG
	>xyl_r	ATG ATT GGA TCG ACC TCA GC
cpCELL	>cell_f	GCC TCC AGA CCC ATT TC TTT
	>cell_r	CAT CGA AGA TGG TGA CAA CG
cpXTH	>xth_f	GCA CTC AGG AGG AAG AGT A
	>xth_r	GTG GGA CCA GAA GGA GTT T
cpACT	>act_f	CGT GAC CTT ACT GAT CAC TTG
	>act_r	GTC AAG GGC AAT GTA AGA CAG
cpEF1	>ef1_f	GTT AAG AAC GTT GCC GTG AAG
	>ef1_r	ATG TGA AGT TGG CTG CTT CCT
cpUBQ	>ubq_f	ACT CAC CGG CAA GAC CAT
	>ubq_r	GTG GAG AGT CGA TTC CTT TTG

**Table S2. GenBank Annotations**
PG1: FJ007644
PG2: GQ479791
PG3: GQ479794
PL1: DQ660903
PL2: ABIM01001816
AGAL1: ABIM01008846
AGAL3: ABIM01016598
BGAL1: AF064786
BGAL3: ABIM01026480
PME1: GR486204
PME2: ABIM01018702
PME3: ABIM01014785
ARF: GQ479793
XYL: AY138968
CELL: ABIM01009161
XTH: ABIM01014233

Table S3. Calibration curves for relative gene expression.

Name of the gene	*Efficiency (10-slope)*	*y=ax+b*	*R^2*
cpPG1	1.96	-3.4408x + 22.295	0.9973
cpPG2	1.94	-3.4841x + 27.584	0.9906
cpPG3	1.93	-3.4997x + 28.439	0.9999
cpPL1	1.98	-3.3722x + 23.508	0.9954
cpPL2	1.82	-3.8546x + 35.543	0.9922
cpAGAL1	2.20	-2.9239x + 25.249	0.9872
cpAGAL3	2.17	-2.9673x + 25.612	0.9689
cpBGAL1	2.05	-3.2145x + 24.034	0.9895
cpBGAL3	1.93	-3.4933x + 25.775	0.9992
cpPME1	1.95	-3.4408x + 22.295	0.9993
cpPME2	1.94	-3.4841x + 27.584	0.9906
cpPME3	1.93	-3.4997x + 28.439	0.9999
cpARF	2.00	-3.3321x + 27.523	0.9885
cpXYL	1.94	-3.4742x + 23.389	0.9998
cpCELL	1.86	-3.7239x + 24.695	0.9942
cpXTH	1.84	-4.3541x + 29.412	0.9785
cpACT	2.03	-3.2446x + 21.922	0.9884
cpEF1	1.92	-3.5886x + 25.540	0.9779
cpUBQ	1.89	-3.4841x + 22.049	0.9906
